# Retrospective Analysis of Feline Leukemia Virus (FeLV) Frequency in Domestic Cats in Quito, Ecuador (2021–2024)

**DOI:** 10.3390/ani15101469

**Published:** 2025-05-19

**Authors:** Byron Puga-Torres, Hugo Navarrete, David de la Torre

**Affiliations:** 1 LABIGEN, Laboratorio de Biología y Genética Molecular, Quito 170133, Ecuador; 2 Centro de Estudios Aplicados en Química (CESAQ-PUCE), Escuela de Ciencias Biológicas, Pontificia Universidad Católica del Ecuador, Quito 170525, Ecuador; 3 IRBS, Institute for Research in Biological Sciences, Quito 170133, Ecuador; direccion@irbsfoundation.org

**Keywords:** cats, prevalence, Ecuador, feline leukemia, RT-PCR

## Abstract

Feline leukemia (FeLV) is a viral disease that primarily affects domestic cats (Felis catus), with high mortality rates because it mainly targets the immune system and is also associated with lymphomas. The objective of this study is to retrospectively analyze the prevalence of FeLV in cats treated at veterinary centers in the city of Quito, between September 2021 and December 2024. Data were obtained from diagnostic test results conducted at the Laboratory of Biology and Molecular Genetics (LABIGEN) using RT-qPCR. A total of 850 samples met the inclusion criteria. FeLV was detected in 28.59% (243/850) of samples, with a slightly higher prevalence in males (53.50%) than females (46.50%). Regarding age, 54.32% (132/243) were between 1 and 5 years, 22.22% (54/243) were between 1 and 11 months, 18.52% (45/243) were between 5 and 10 years, and 4.94% (12/243) were between 10 and 19 years. In conclusion, the prevalence of FeLV in Quito, Ecuador, is high, requiring greater efforts to prevent and control this disease, in pursuit of animal health and well-being.

## Simple Summary

Feline leukemia is a viral disease in cats that has a high mortality rate because it affects their immune system. We reviewed the results of laboratory tests conducted in Quito, Ecuador, between September 2021 and December 2024, finding the disease in 28.59% (243/850) of cases, with a higher prevalence in animals aged 1 to 5 years. We conclude that the prevalence of the disease is high in Quito, Ecuador, and this requires the implementation of prevention and control measures for this infection.

## 1. Introduction

Feline leukemia (FeLV) is a disease caused by a virus from the *Retroviridae* family, a group of RNA viruses that use reverse transcriptase to integrate into the host’s DNA. This enables the virus to integrate into the cellular genome, ensuring its persistence and facilitating vertical and horizontal transmission. It mainly affects domestic cats (*Felis catus*), although it has also been detected in some wild cat populations, and it is considered one of the main causes of death due to its devastating effects on the immune system and its potential to be accompanied by hematological and non-hematological neoplasms [1,2,3]. In non-domestic cats, the disease has been identified in animals from South and Central America, for example, in the jaguarundi (*Puma yagouaroundi)*, as well as in free-roaming pumas (*Puma concolor*) [4,5].

FeLV remains a clinical and epidemiological challenge due to its high transmission rate and the lack of curative preventive treatments. Its infectious nature and persistence in uncontrolled populations highlight the need to implement effective diagnostic and management programs, especially for pet cats living outdoors, strays, and shelter cats, without neglecting those that are domestic [6].

A distinctive feature of FeLV is its capacity to generate genetic variants via recombination and mutation, leading to four main subgroups: A, B, C, and T. Subgroup A is known to be necessary for primary infection, whereas the other subgroups are linked to specific complications such as severe anemia and lymphomas [6,7,8]. Transmission of FeLV primarily occurs through fluids such as saliva, tears, and maternal milk, which explains why high-density feline communities—such as shelters and street colonies—present higher infection rates [6,7,8]. It is important to note that not all cats exposed to FeLV develop active disease, since this largely depends on the host’s immune response. In some cases, the infection may be transient, while in others, the virus establishes a persistent or latent infection [2,9].

The symptomatology of FeLV varies greatly depending on the stage of the infection and the infected cat’s immune system. Initially, cats may remain asymptomatic or present non-specific signs such as lethargy, mild fever, and loss of appetite, which may go unnoticed [6,8], leading one to believe that the cat has recovered. Conversely, in persistent infections, clinical signs become more severe and include anemia, jaundice, and generalized lymphadenopathy. Additionally, patients may develop secondary infections such as stomatitis, respiratory infections, and chronic diarrhea due to FeLV-induced immunosuppression. In advanced stages, severe complications such as lymphomas, leukemias, and degenerative diseases are observed [2,10].

An efficient control measure for cats with a confirmed diagnosis of FeLV involves identifying and isolating the animals. Moreover, sterilization significantly improves the life expectancy of infected cats [11,12]. In preventive management, vaccination remains the most effective strategy to reduce FeLV incidence. Inactivated and recombinant vaccines are available and recommended, particularly for cats at risk, such as those in contact with infected felines or living in street colonies. However, vaccination is not completely foolproof—no vaccine confers absolute protection, and they are not useful for cats already infected—highlighting the importance of preliminary diagnostic tests, mainly based on the detection of the p27 antigen through enzyme-linked immunosorbent assay (ELISA) or immunochromatographic (Snap Test) techniques, which are rapid, accessible, and effective for identifying active infections in early stages [2,8,13].

It should be noted that test sensitivity can vary depending on an individual’s viral load, which can result in an underestimation of disease prevalence, less vigilant control, and, consequently, increased risk of transmission among cats that are actually diseased. To enhance the accuracy of the diagnosis, molecular tests such as polymerase chain reaction (PCR) are mandatory. PCR can detect proviral DNA in cats with latent infections or low viral replication, making it particularly useful for differentiating transient from persistent infections—an important factor in clinical management [8,14,15,16]. Furthermore, recent studies suggest that p27 antigen concentrations may correlate with disease severity, offering an additional marker for evaluating prognosis [2].

With this background in mind, and given the limited prevalence studies on FeLV in Quito, Ecuador, the present investigation aimed to determine the prevalence of this disease in cats from Quito by applying RT-qPCR at the Laboratory of Biology and Molecular Genetics (LABIGEN). These animals were treated at veterinary centers in the city between September 2021 and December 2024. 

## 2. Materials and Methods

### 2.1. Sampling Area and Study

This is an observational, retrospective study based on laboratory analyses conducted between September 2021 and December 2024, on animals treated at veterinary centers in Quito, Ecuador. Inclusion criteria were laboratory reports that included the patient’s sex, date of birth, test result, and sample type. Records that did not contain all this information were eliminated.

A total of 850 cat analyses met the inclusion criteria and were evaluated, which included information on sex, date of birth of the patient, test results, and the type of sample obtained. The age data were subdivided into four categories: 1 month–11 months old, 1–5 years old, 5–10 years old, and 10–19 years old. The sample types analyzed included whole blood (with EDTA anticoagulant), whole blood plus swab (saliva swab), serum (from clotted blood), and serum plus whole blood, to detect the presence of the virus in the samples. The swabs used were Sterile Polyester Tipped Applicators (Puritan Medical Products—31 School St. P.O. Box 149, Guilford, ME, USA)

### 2.2. Molecular Analysis

The protocol for nucleic acid extraction was based on the manufacturer’s instructions for the Mag MAX™ CORE Nucleic Acid Purification Kit (catalog A32702—Applied Biosystems™—ThermoFisher Scientific, Waltham, MA, USA). To detect the presence of the virus, the RT-qPCR technique (equipment QuantStudio 3—Applied biosystems) was used at the Laboratory of Biology and Molecular Genetics (LABIGEN, Quito, Ecuador), a reference laboratory in Ecuador. The protocol detailed by Velilla et al. (2020), “Standardization of real-time multiplex PCR for the diagnosis of AIDS and leukemia in Felis silvestris catus”, was followed [17].

This multiplex PCR technique used TaqMan probes, which amplify a constitutive gene present in the cat genome, ensuring the reliability of the results obtained. To detect feline leukemia virus (FEV), the following primers were used: sense primer (5′–3′) TATTGGGCCTGTAACACTG, antisense primer (5′–3′) GACTTACCATCAACCCGAA, and probe (5′–3′) TTTCCATGGCGGTGCTCAATTGGA.

RNA extraction was performed using the GeneJET RNA Purification kit (Thermo Scientific, Inc., Waltham, MA, USA), following the manufacturer’s instructions. A total of 200 µL of peripheral blood was used, which was subjected to cell lysis with β-mercaptoethanol, followed by purification using silica membrane columns and washing solution, and finalized with an elution step. The obtained RNA was stored at −20 °C. Complementary DNA (cDNA) was then synthesized from the RNA using the First Strand cDNA Synthesis Kit (Thermo Scientific, Inc., Waltham, MA, USA), using non-specific primers provided in the kit, and also following the manufacturer’s instructions.

For PCR, the QuantiTect Multiplex PCR NoROX Kit (QIAGEN Aarhus, Aarhus, Denmark) was used, and detection was performed using TaqMan probes in a real-time thermal cycler. Negative controls (without cDNA) were included to establish the detection threshold in each amplification channel, calculated with a 95% confidence interval. Based on this threshold, samples were judged to be positive or negative. The multiplex PCR was optimized with the following conditions: 12.5 µL of QuantiTect Multiplex PCR NoROX Master Mix 2x, 0.67 µL of each primer and probe at 10 mM, a cDNA concentration equal to or greater than 10,000 ng for the reaction, and RNase-free water to bring the final volume to 25 µL. The amplification program consisted of an initial denaturation at 94 °C for 15 min, followed by 50 cycles with the following steps: 94 °C for 60 s (primer annealing), 59 °C for 60 s (probe annealing), and 68 °C for 35 s (elongation).

To interpret the multiplex PCR results, any sample that exceeded the detection threshold in at least one of the virus-specific channels and the internal control was considered positive. An event was classified as negative if it only exceeded the threshold in the internal control channel and as undetermined if the threshold was not exceeded in any of the channels.

### 2.3. Statistical Analysis

Prevalence was defined as the total number of positive samples relative to the total number of samples tested. A database was constructed using laboratory reports from LABIGEN, transferred to a Microsoft Excel spreadsheet. Frequency tables were generated for FeLV-positive animals based on sex, age, and sample type. Statistical comparisons were performed using the chi-square test (χ^2^) at a 0.05 significance level with RCran software version 1.2.5019 (RStudio Inc., Boston, MA, USA) and its RStudio platform version 2024.04.2+764 to compare disease positivity, feline sex, age, and sample type.

## 3. Results

### 3.1. General Data

Between September 2021 and December 2024, a total of 876 analyses were conducted, of which 850 met the established inclusion criteria. It should be noted that the patients were treated at different veterinary centers in the city of Quito. Regarding sex, out of the 850 samples, 52.71% were from females and 47.29% from males. Ages ranged from 1 month to 229 months (19 years and 1 month), with a mean age of 42 months (3 years and 6 months). Regarding the presence of the virus in the samples, RT-qPCR determined that 243 patients (28.59%) tested positive for the virus, while 607 patients (71.41%) tested negative for the feline leukemia virus (Table 1).

### 3.2. FeLV-Positive Animals

Out of the 243 animals that tested positive, 53.50% (130/243) were male and 46.50% (113/243) were female (Table 2), representing a statistically significant difference (*p* = 0.03). Regarding the ages of FeLV-positive animals, 54.32% (132/243) were cats between 1 and 5 years old, 22.22% (54/243) were between 1 month and 11 months, 18.52% (45/243) between 5 and 10 years, and 4.94% (12/243) between 10 and 19 years (Table 2), with no statistically significant differences among age groups (*p* = 0.22). Regarding sample type, of the 243 FeLV-positive cases, 62.55% (152/243) were whole blood, 36.21% (88/243) were whole blood plus swab, 0.82% (2/243) were serum, and 0.41% (1/243) were serum plus blood (Table 2), with no statistically significant differences among sample types (*p* = 0.11).

## 4. Discussion

Few studies have reported on the prevalence of FeLV in Ecuador. Our results show that 28.59% (243) of the evaluated animals tested positive for the feline leukemia virus. This figure is higher than the 20.3% reported by [18] in a study of 384 domestic cats in Quito (random sampling) using rapid immunochromatographic tests on blood samples. Likewise, retrospective data from 2013 to 2018, based on 321 domestic cats treated in three veterinary centers in Quito, indicated a feline leukemia prevalence of 16.82% [19].

Comparisons with other cities in Ecuador reveal a high degree of heterogeneity. For example, in urban areas of the Montalvo canton, Los Ríos Province, using immunochromatographic methods on domestic animals, an FeLV prevalence of 6.18% (6/97) was found [20]. Similarly, in Fátima Parish, Pastaza Province, the prevalence was 7.44% (9/121) in the domestic cats tested [21], whereas in Cuenca City, based on samples analyzed at the veterinary clinic of the Salesian Polytechnic University, prevalence reached 34% among 100 animals tested [22]. Much higher rates were reported in Santo Domingo de los Tsáchilas Province, where 68.1% of 47 cats treated at a local veterinary clinic tested positive [23].

In Colombia, in the city of Tunja, a convenience sampling study spanning over six months in a local veterinary clinic reported an FeLV prevalence of 17% [24]. Similarly, in the Risaralda department of Colombia, a prevalence of 24.3% was found among 308 animals tested [25]. In Maracaibo, Venezuela, a study of shelter cats found a virus positivity of 2.1% (95) [26]. In Brazil, various studies have also shown differing FeLV prevalence rates, ranging from just over 20% in urban cats facing challenging environmental conditions and limited veterinary care access [6], to 45.6% in 384 blood samples from cats receiving hospital care, because the usual activities of these cats make them more prone and at greater risk of contracting the virus [27]. In 274 cats treated at the Veterinary Hospital of the State University of Santa Catarina (UDESC), Brazil, 28.41% tested positive [28]. Meanwhile, in 1322 stray and owned cats from the Campania, Basilicata, and Calabria regions of Italy over 10 years (using ELISA), 7.64% tested positive for feline leukemia [29].

Prevalence also varies across European countries, with low values such as 2.7% in Switzerland [30], and in Hungary, where in 335 blood samples from domestic cats tested via ELISA and PCR, FeLV prevalence was 11.8% and 17.3%, respectively—considered relatively high [31]. A multi-country study in Europe utilizing ELISA and qPCR reported the following prevalence rates: Italy 21.2%, Portugal 20.4%, Germany 9.5%, and France 9.3%, with higher rates in dense urban populations and among stray cats [8]. In cats from two rehoming centers in the UK (between August 2011 and August 2012), FeLV prevalence was 3% (14/473) in one center, while none was found among the 135 animals in the other. All these data confirm that FeLV infection varies considerably between locations within the same country or among neighboring countries [32]. Furthermore, in Southeast Asia, a study from 2017 to 2018 reported that 18.5% of 119 cats in Thailand tested positive, whereas none of the 45 cats in Indonesia did. In Canada, a retrospective study in both a primary care hospital and a referral hospital found a prevalence of 4.3% among 1692 animals [14].

Regarding the sex of FeLV-positive animals in our study, 53.50% (130/243) were males and 46.50% (113/243) were females—similar to findings from Cuenca (Ecuador), where 58.82% of infected animals were males and 41.18% were females [22]. In contrast, in Fátima Parish, Pastaza Province, Ecuador, Pérez & Borja (2023) found no differences between sexes [21]. Outside Ecuador, a Canadian study yielded similar findings to our own, with 56.16% (41/73) of FeLV-infected cats being male and 43.84% (32/73) female [14]. Similarly, in Risaralda, Colombia, the infection was also higher in males than females [25], as was the case in Brazil [27]. Male cats are more susceptible because they tend to spend much more time outdoors, as well as due to fights with other cats, and because they can be infected by females with the disease [33,34].

In terms of the age of infected cats, more than half in our study were between 1 and 5 years old. This aligns with findings from Cuenca, Ecuador, where most infected animals (50%) were older than 12 months [22], while in Fátima Parish, Pastaza Province, there were no significant age differences [21]. These results also match observations from Canada, where the average age of infected animals was 3.1 years [14], and from Brazil, where around 50% of infected cats were young [31], or the average age was 3 years in another study [28]. It is quite common to associate the disease with young cats, as they can acquire the disease from their mother, either through placental union with fetus or during lactation (through milk). Another risk factor is that young cats are more likely to explore the outdoors and even share food bowls with other infected cats. Additionally, young cats tend to receive the least veterinary care [33,34].

FeLV represents a significant challenge for both cat owners and the veterinary community. As a retrovirus with a high capacity for mutation and recombination, its genetic variability and transmission mechanisms have hampered effective disease control, particularly in stray cat populations and in regions with limited access to veterinary services, and there is no cure for the disease [3,6]. The findings of this study, which determine a high prevalence of the disease in Quito, Ecuador, emphasize the importance of early diagnosis to improve the prognosis of infected cats and prevent the spread of the disease, as well as the use of vaccination, although it should be accompanied by isolation and health control measures [2,8]. Additionally, management programs such as the trap–neuter–return method have been shown to be effective in reducing the prevalence of FeLV in feral cat colonies, as demonstrated by a study conducted in Switzerland [8]. Awareness and education campaigns have emerged as valuable tools to reduce prevalence, encourage vaccination, and promote isolation of infected cats [3]. Furthermore, a well-structured adoption program for infected animals, supported by ongoing post-adoption support, can significantly extend the life expectancy of these cats [35].

## 5. Conclusions

In conclusion, an FeLV prevalence of 28.59% (243/850) was found among animals treated at several veterinary centers in Quito, Ecuador, between September 2021 and December 2024. This relatively high prevalence predominantly affected adult male cats between 1 and 5 years old. Although significant advancements have been made, FeLV remains a disease with global impact on feline health. In Ecuador, future research should focus on improving vaccine efficacy, developing more accessible therapies, and deepening our understanding of the genetic and environmental factors that influence susceptibility and disease progression. Only through a comprehensive approach—combining science, technology, and education—can we mitigate FeLV’s devastating consequences in feline populations. Likewise, confirmatory tests (such as PCR) should be mandatory to minimize false negative diagnoses in Ecuador’s feline population.

## Figures and Tables

**Table 1:** Sex, age, and results of all cats included in the study.

Variable	Category	Number	Percentage
Sex	Male	448	52.71%
	Female	402	47.29%
Age	1–11 months	201	23.65%
	1–5 years	419	49.29%
	5–10 years	171	20.12%
	10–19 years	59	6.94%
FeLV Status	Negative	607	71.41%
	Positive	243	28.59%

**Table 2:** Sex, age, and results of FeLV-positive cats.

Variable	Category	Number	Percentage	*p*-Value
Sex	Male	130/243	53.50%	0.03 *
	Female	113/243	46.50%	
Age	1–11 months	54/243	22.22%	0.22
	1–5 years	132/243	54.32%	
	5–10 years	45/243	18.52%	
	10–19 years	12/243	4.94%	
Sample	Whole blood	152/243	62.55%	0.11
	Whole blood + swab	88/243	36.21%	
	Serum	2/243	0.82%	
	Serum + blood	1/243	0.41%	

* Statistically significant differences.

## Data Availability

The data will be made available upon reasonable request.
